# Identification of targetable kinases in idiopathic pulmonary fibrosis

**DOI:** 10.1186/s12931-022-01940-y

**Published:** 2022-02-07

**Authors:** Hisao Higo, Kadoaki Ohashi, Shuta Tomida, Sachi Okawa, Hiromasa Yamamoto, Seiichiro Sugimoto, Satoru Senoo, Go Makimoto, Kiichiro Ninomiya, Takamasa Nakasuka, Kazuya Nishii, Akihiko Taniguchi, Toshio Kubo, Eiki Ichihara, Katsuyuki Hotta, Nobuaki Miyahara, Yoshinobu Maeda, Shinichi Toyooka, Katsuyuki Kiura

**Affiliations:** 1 Department of Hematology, Oncology and Respiratory Medicine, Dentistry and Pharmaceutical Sciences, Okayama University Graduate School of Medicine, Okayama, Japan; 2 Department of Respiratory Medicine, Okayama University Hospital, 2-5-1 Shikata-cho, Kita-ku, Okayama, 700-8558 Japan; 3 Center for Comprehensive Genomic Medicine, Okayama University Hospital, Okayama, Japan; 4 Department of Thoracic Surgery, Okayama University Hospital, Okayama, Japan; 5 Organ Transplant Center, Okayama University Hospital, Okayama, Japan; 6 Center for Clinical Oncology, Okayama University Hospital, Okayama, Japan; 7 Center for Innovative Clinical Medicine, Okayama University Hospital, Okayama, Japan; 8 Department of Medical Technology, Okayama University Graduate School of Health Sciences, Okayama, Japan; 9 Department of General Thoracic Surgery and Breast and Endocrinological Surgery, Dentistry and Pharmaceutical Sciences, Okayama University Graduate School of Medicine, Okayama, Japan

**Keywords:** Idiopathic pulmonary fibrosis, RNA sequencing, Molecular therapeutic target, Personalized therapy

## Abstract

**Background:**

Tyrosine kinase activation plays an important role in the progression of pulmonary fibrosis. In this study, we analyzed the expression of 612 kinase-coding and cancer-related genes using next-generation sequencing to identify potential therapeutic targets for idiopathic pulmonary fibrosis (IPF).

**Methods:**

Thirteen samples from five patients with IPF (Cases 1–5) and eight samples from four patients without IPF (control) were included in this study. Six of the thirteen samples were obtained from different lung segments of a single patient who underwent bilateral pneumonectomy. Gene expression analysis of IPF lung tissue samples (n = 13) and control samples (n = 8) was performed using SureSelect RNA Human Kinome Kit. The expression of the selected genes was further confirmed at the protein level by immunohistochemistry (IHC).

**Results:**

Gene expression analysis revealed a correlation between the gene expression signatures and the degree of fibrosis, as assessed by Ashcroft score. In addition, the expression analysis indicated a stronger heterogeneity among the IPF lung samples than among the control lung samples. In the integrated analysis of the 21 samples, *DCLK1* and *STK33* were found to be upregulated in IPF lung samples compared to control lung samples. However, the top most upregulated genes were distinct in individual cases. *DCLK1*, *PDK4*, and *ERBB4* were upregulated in IPF case 1, whereas *STK33*, *PIM2*, and *SYK* were upregulated in IPF case 2. IHC revealed that these proteins were expressed in the epithelial layer of the fibrotic lesions.

**Conclusions:**

We performed a comprehensive kinase expression analysis to explore the potential therapeutic targets for IPF. We found that DCLK1 and STK33 may serve as potential candidate targets for molecular targeted therapy of IPF. In addition, PDK4, ERBB4, PIM2, and SYK might also serve as personalized therapeutic targets of IPF. Additional large-scale studies are warranted to develop personalized therapies for patients with IPF.

**Supplementary Information:**

The online version contains supplementary material available at 10.1186/s12931-022-01940-y.

## Background

Idiopathic pulmonary fibrosis (IPF) is a chronic, progressive lung disease that results in fibrotic scarring of the alveolar tissues. Globally, the incidence of IPF is increasing, with approximately 3–9 cases per 100,000 individuals being reported each year [1]. Anti-fibrotic drugs, such as pirfenidone and nintedanib, which suppress disease progression, have been clinically approved for the treatment of IPF [2–5]. However, the overall survival of IPF patients is low, ranging from 2 to 3 years [6, 7]. Although a few patients with IPF and severe respiratory failure have been treated with lung transplantation [8, 9], strict eligibility criteria and shortage of organ donors often limits transplantation therapy.

Multiple studies have shown that damage to the respiratory epithelium and impairment in its repair mechanism play a central role in the development of IPF [10]. In particular, alveolar type II (AT2) cells play important roles in the pathogenesis of IPF because they act as progenitor cells and help in regeneration of the respiratory epithelium [11, 12]. Currently, multiple gene mutations affecting the function or survival of AT2 cells have been reported in IPF lung tissues [13, 14]. In addition, single nucleotide polymorphisms in mucin 5B (MUC5B), resulting in the abnormal production of mucin, are known to play a role in IPF pathogenesis [15]. Furthermore, the incidence of IPF increases with aging, which suggests the existence of a complex relationship between chronic environmental exposure, infection, host defense/repair pathways, and disease progression.

Currently, there are multiple clinically approved kinase inhibitors for a wide range of diseases, including fibrosis and malignant diseases. Furthermore, there are a few similarities in the pathogenesis of IPF and non-small cell lung cancer (NSCLC), a chronic respiratory disease with abnormal cell proliferation. In addition, the activation of tyrosine kinases and overexpression of growth factors have been known to play important roles in the progression of both pulmonary fibrosis [16, 17] and lung cancers [18]. Nintedanib has the potential to inhibit the activity of multiple kinases, including vascular endothelial growth factor receptor, fibroblast growth factor receptor, and platelet-derived growth factor receptor [19]. Interestingly, nintedanib has also demonstrated a beneficial effect on tumor suppression in clinical trials involving patients with advanced NSCLC [20]. Based on these findings, we hypothesized that other kinase inhibitors may also have the potential to inhibit the progression of IPF.

In this study, we analyzed the expression of 612 kinase and cancer-related genes to identify the potential therapeutic targets of IPF. We used next-generation sequencing to perform gene expression analysis of 13 and 8 surgically resected lung tissues from patients with and without IPF, respectively. Further, we validated the expression of selected genes at the protein level in fibrotic lesions using immunostaining.

## Methods

### Patients and sample preparation

This study (registered number: K1505-033) was approved by the Ethics Committee of Okayama University Graduate School of Medicine, Dentistry and Pharmaceutical Sciences and Okayama University Hospital.

Patients with or without IPF were enrolled in this study between April 2015 and November 2016 after obtaining written informed consent. The tissue samples with or without IPF were obtained from the organs removed for lung transplantation or resected during the treatment of lung cancer at Okayama University Hospital. The diagnosis of IPF was based on the official ATS/ERS/JRS/ALAT statement [21]. The collected samples were immediately cut into small sections and fixed using PAXgene^Ⓡ^ Tissue System (PreAnalytiX, Hombrechtikon, Switzerland) or RNAlater™ (Sigma-Aldrich, St. Louis, MO, USA). RNA was extracted from RNAlater™-fixed samples using the RNeasy Micro Kit according to the manufacturer’s protocol. The concentration and quality of RNA were measured using an Agilent 2100 Bioanalyzer (Agilent Technologies, Santa Clara, CA, USA).

### Targeted RNA sequencing and data analysis

The SureSelect RNA Human Kinome Kit (Agilent Technologies) that targets 612 genes, including 517 protein kinase-coding genes and 46 cancer-related genes, was utilized for library preparation using the RNA samples with an RNA integrity number > 7. Sequencing was performed on an Illumina MiSeq Sequencing System using the V2 Reagent Kit (Illumina, San Diego, CA, USA). The sequencing data were analyzed using the CLC Genomics Workbench (CLC bio, Aarhus, Denmark). Gene expression data were normalized based on the reads per kilobase per million mapped reads (RPKM). Hierarchical clustering analysis was performed using Cluster 3.0 software with the following adjusting method: centralization with median value of each sample, and normalization by dividing centered values by the standard deviation of all samples. Gene expression diversity was calculated with the following adjusting method: the difference between the maximum and minimum RPKM values was calculated for each gene in each patient, and the obtained values were divided by the standard deviation of all samples.

### Histological analysis

The paraffin-embedded tissue blocks fixed using PAXgene^Ⓡ^ Tissue System were cut into 5-µm thick sections and stained with hematoxylin and eosin. The severity of fibrosis in these samples was evaluated using the Ashcroft score, as previously described [22].

### Immunohistochemistry (IHC)

The paraffin-embedded tissue blocks fixed using PAXgene^Ⓡ^ Tissue System were cut into 5 µm thick sections, placed on glass slides, and deparaffinized in d-limonene and graded alcohol. The tissue sections were then incubated in 1 mM EDTA buffer (pH 8.0) for 10 min at 95 °C in a water bath and blocked for endogenous peroxidase activity with 3% hydrogen peroxide in methanol for 5 min. Following the incubation, the slides were rinsed with Tris-buffered saline containing 0.1% Tween 20 and blocked with normal goat serum or normal horse serum for 60 min. The sections were then incubated with primary antibodies overnight at 4 °C. The primary antibodies against doublecortin-like kinase 1 (DCLK1) (ab31704), pyruvate dehydrogenase kinase 4 (PDK4; ab71240), and spleen-associated tyrosine kinase (SYK; ab40781) were obtained from Abcam (Cambridge, MA, USA). Anti-Pim-2 proto-oncogene, serine/threonine kinase (anti-PIM2; HPA000285) and anti-serine/threonine kinase 33 (anti-STK33; HPA015742) antibodies were purchased from Sigma-Aldrich (St. Louis, MO, USA), and anti-Erb-B2 receptor tyrosine kinase 4 (anti-ERBB4) antibody (19943-1-AP) was obtained from Proteintech Japan (Tokyo, Japan). Following overnight incubation, the sections were incubated with EnVision + Single Reagents HRP Rabbit (Dako, Glostrup, Denmark) or ImmPRESS Reagent Anti-Mouse IgG (Vector Laboratories, Burlingame, CA, USA) secondary antibodies for 20 min. Finally, the sections were stained with 3,3-diaminobenzidine and counterstained with hematoxylin.

### Signal-to-noise weighted-voting score

Samples of IPF case 1 were used to select the genes for weighted-voting score calculation. Samples of IPF cases 2 and 3 were used as an independent validation set to evaluate the model versatility. The signal-to-noise statistic (S_x_) was calculated as described previously [23, 24]. Briefly, the signal-to-noise statistic (S_x_) is calculated as the weight for gene x as S_x_ = (μ_Ashcroft≥6_ − μ_Ashcroft<6_/σ_Ashcroft≥6_ + σ_Ashcroft<6_), where, for each gene, μ_Ashcroft≥6_ represents the mean value and σ_Ashcroft≥6_ represents the standard deviation for that gene in all samples of the Ashcroft score ≥ 6. In all, 26 genes were selected as “induced” genes showing (μ_Ashcroft≥6_ − μ_Ashcroft<6_) > 1, whereas 15 genes showing (μ_Ashcroftt≥6_ − μ_Ashcroft<6_) < -1 were selected as “suppressed” genes in Ashcroft score ≥ 6 samples (Additional file 1: Table S1). A weighted-voting classification algorithm was employed to predict Ashcroft score ≥ 6 and/or Ashcroft score < 6 samples using the genes selected as described above, and the resulting classifiers were tested using the independent dataset (IPF cases 2 and 3). “Weights” were calculated based on triplicated Ashcroft score ≥ 6 samples and triplicated Ashcroft score < 6 samples of case 1. In this scheme, gene x of a test sample γ in the predictive gene set has a vote based on its expression in this sample (g_x_^γ^) using weight S_x_, boundaries b_x_ = (μ_Ashcroft≥6_ + μ_Ashcroft<6_)/2, and weighted voting score V_x_ = S_x_ (g_x_^γ^ − b_x_). The final voting scores were summed (∑_x_V_x_).

### Statistical analysis

Statistical analyses were performed using STATA software version 15.1 (StataCorp, College Station, TX, USA). Expression of targetable kinase genes between the individual case and the control lung samples were compared using two-tailed paired Student’s t-tests.

## Results

### Characteristics of IPF and control patients

The characteristics of five IPF and four control patients are shown in Table 1. The median age of IPF patients was 57 years (range, 56–67 years). IPF lung tissues were harvested from two patients, who underwent lung transplantation, and from three patients, who underwent surgical lung resection for NSCLC. Multiple samples (2–6 samples) were obtained from three IPF patients (cases 1, 2, and 3), whereas a single sample was collected from each of the remaining two patients (cases 4 and 5). Thus, in total, 13 lung tissue samples were obtained from five IPF patients. The median age of the control patients was 64 years (range, 45–82 years). The lung tissues without IPF were harvested from the control patients who underwent surgical lung resection for NSCLC. A total of eight lung tissue samples were collected from four control patients (two from each patient).

**Table 1:** Patient characteristics and number of obtained samples

IPF	Age (years)	Sex	Smoking history (pack-years)	Time from IPF diagnosis to surgery	IPF treatment	Lung cancer type	Gene analysis of lung cancer	Surgical procedure	Number of samples
Case 1	57	Male	132	7 years	None	SCC*	NA	Lung transplantation	6
Case 2	67	Male	23.5	4 years	None	SCC	EGFR-	Lobectomy	3
Case 3	61	Male	82.5	5 years	Pirfenidone	–	NA	Lung transplantation	2
Case 4	56	Male	82.5	3 months	None	SCC	NA	Lobectomy	1
Case 5	56	Male	36	3 weeks	None	SCC	NA	Lobectomy	1
*Control*									
Case 1	45	Male	15	–	–	ADC	EGFR Ex18 G719S	Lobectomy	2
Case 2	82	Male	45	–	–	SCC	NA	Lobectomy	2
Case 3	65	Female	22.5	–	–	ADC	KRAS Q61H	Lobectomy	2
Case 4	63	Female	0	–	–	ADC	EGFR-	Lobectomy	2

*IPF* idiopathic pulmonary fibrosis, *SCC* squamous cell carcinoma, *ADC* adenocarcinoma, *EGFR* epidermal growth factor receptor, *KRAS* Kirsten rat sarcoma viral oncogene homolog, *NA* not available

^*^Lobectomy was performed seven years prior to lung transplantation

### Gene expression signatures are altered with the progression of fibrosis

**Fig. 1:**
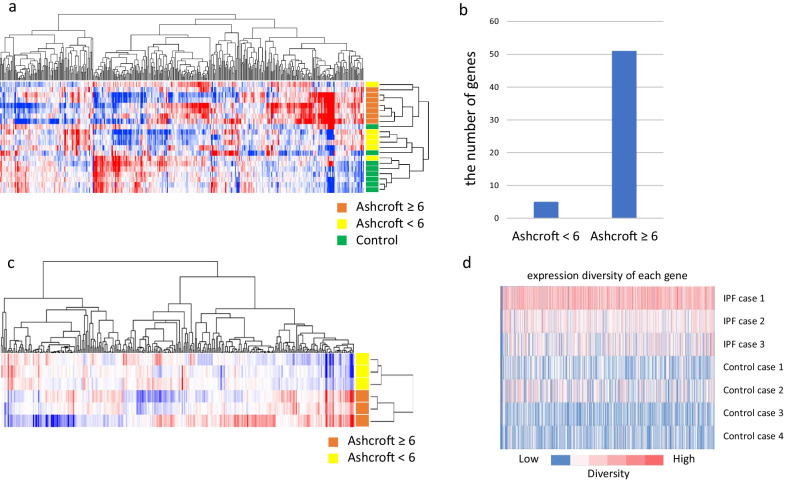
Expression analysis of 612 kinase-coding and cancer-related genes in IPF and control lung samples. **a** Clustering analysis of all samples. **b** Number of genes differentially expressed by more than twofold in moderate and severe fibrotic samples compared to control lung samples. **c** Clustering analysis of six samples from IPF case 1. **d** Gene expression diversity in IPF and control lung samples from each patient. IPF cases 4 and 5 were excluded from the gene expression diversity analysis as they contributed one sample each

Gene expression analysis was performed using 13 IPF and 8 control (without IPF) lung tissue samples. Clustering analysis indicated that the IPF and control samples were clustered together (Fig. 1A). Hence, considering that the severity of fibrosis may affect gene expression, we divided the IPF tissue samples into two subgroups based on the Ashcroft score that estimates the severity of pulmonary fibrosis on a numerical scale (Ashcroft score < 6: normal to moderate fibrosis; Ashcroft score ≥ 6: severe fibrosis) [22]. As expected, the severe fibrotic samples (Ashcroft score ≥ 6) showed an independent gene signature compared to the moderate fibrotic (Ashcroft score < 6) and control samples, whereas the gene expression signature of the moderate fibrotic samples (Ashcroft score < 6) was not independent of that of the control lung samples (Fig. 1a). Additionally, only five genes were found to be differentially expressed by more than twofold in moderate fibrotic samples compared to the control samples, whereas 51 genes were differentially expressed by more than twofold in severe fibrotic samples compared to the control samples (Fig. 1b, Additional file 1: Table S2). Moreover, we performed an independent clustering analysis of the six IPF samples obtained from a single patient (case 1) and confirmed the correlation between the gene expression signature and Ashcroft score (Fig. 1c). Clustering analysis confirmed the correlation of gene expression signature and Ashcroft score in IPF cases 2 and 3 as well (Additional file 1: Fig. S1a, b). Further, we examined whether the change in gene expression according to the severity of fibrosis in IPF case 1 was also observed in IPF cases 2 and 3. As expected, the signal-to-noise weighted-voting score analysis showed that the gene expression signature in the fibrotic tissues with Ashcroft score ≥ 6 or < 6 in IPF case 1 was reproduced in both IPF cases 2 and 3 (Additional file 2: Fig. S1c). Altogether, these results suggest that the gene expression signatures are altered with the progression of fibrosis.

### Gene expression diversity in IPF lung tissues

As IPF lungs typically present with temporal and spatial heterogeneous histological findings, we assessed the gene expression diversity in IPF lung tissues harvested from different segments in the same patient. Therefore, only patients contributing multiple lung samples were included in this analysis (i.e., IPF cases 1–3 and control cases 1–4). Expectedly, a stronger diversity was observed in IPF lung tissues than in control lung tissues, suggesting not only histological, but also genetic heterogeneity of IPF lungs (Fig. 1d).

### Expression of targetable kinase-coding genes in IPF lung

To explore the targetable kinase-coding genes in IPF lung tissues, we sought to identify genes that were upregulated in IPF lung tissues (n = 13) compared to control lung tissues (without IPF, n = 8) with a fold change of > 2. The integrated analysis indicated three genes (*DCLK1*; *STK33*; and *cyclin-dependent kinase 1*, *CDK1*) to be upregulated with the considered threshold of > twofold in 13 IPF samples compared to the 8 control samples (Table 2).

**Table 2:** Upregulated kinases in idiopathic pulmonary fibrosis compared to control samples

Gene symbol	Gene name	log_2_ ratio
DCLK1	Doublecortin Like Kinase 1	1.78
STK33	Serine/Threonine Kinase 33	1.34
CDK1	Cyclin Dependent Kinase 1	1.10
PDK1	Pyruvate Dehydrogenase Kinase 1	0.85
STK39	Serine/Threonine Kinase 39	0.81
STK16	Serine/Threonine Kinase 16	0.80
PAK1	P21 Activated Kinase 1	0.76
MAPK10	Mitogen-Activated Protein Kinase 10	0.75
MERTK	MER Proto-Oncogene, Tyrosine Kinase	0.71
CDK4	Cyclin Dependent Kinase 4	0.70

Further, considering the heterogeneous nature of IPF, we independently compared the gene expression profiles between the IPF lung samples collected from each of the three cases and control lung. In addition, we evaluated the expression of the 46 selected genes encoding kinases having clinically available kinase inhibitors (Additional file 1: Table S3) [25]. However, of the 46, six genes (*ALK receptor tyrosine kinase*, *Ret proto-oncogene, neurotrophic receptor tyrosine kinase 1*, *neurotrophic receptor tyrosine kinase 3*, *Fms-related tyrosine kinase 3*, and *protein kinase C gamma*) were excluded from further analysis because their RPKM values were lower than the overall median RPKM value. The findings from each of the IPF cases are shown below.

**Fig. 2:**
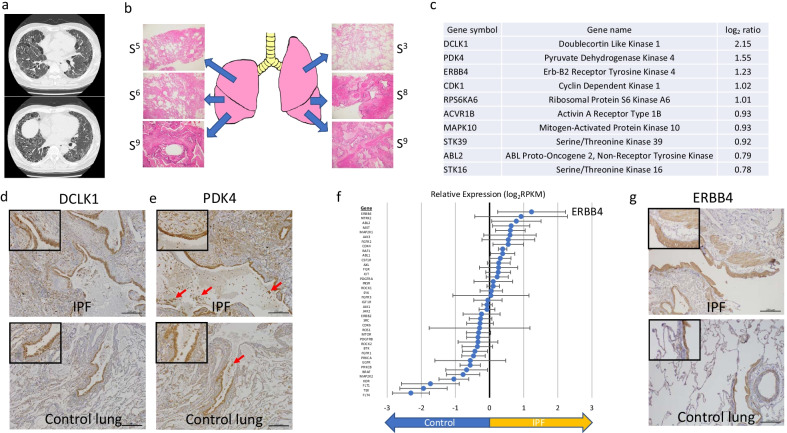
Clinical features and kinase expression profile of IPF lung tissues from case 1. **a** High-resolution computed tomography images of the lung. **b** Sampling site and hematoxylin and eosin staining of each lung tissue. **c** Top 10 kinase-coding genes upregulated in IPF case 1 lung samples compared to control lung samples. **d** Immunohistochemistry of DCLK1. Scale bar = 200 µm. **e** Immunohistochemistry of PDK4. Red arrows indicate alveolar macrophages. Scale bar = 200 µm. **f** Expression profile of the 40 selected genes encoding kinases having clinically available kinase inhibitors. Error bars indicate standard error. **g** Immunohistochemistry of ERBB4. Scale bar = 200 µm

*Case 1:* A 57-year-old male with smoking habit (132 pack-years) and history of right upper lobectomy for squamous NSCLC. High-resolution computed tomography (HRCT) revealed bilateral honeycombing, indicating a usual interstitial pneumonia (UIP) pattern (Fig. 2a). Pathological examination confirmed that the fibrosis was UIP. Based on these findings and exclusion of other causes of pulmonary fibrosis, such as collagen disease, the patient was finally diagnosed with IPF. Lung fibrosis gradually progressed, and total pneumonectomy and bilateral cadaveric lung transplantation were performed. We obtained six lung tissue samples from each of the bilateral lung segments from this patient (Fig. 2b). Figure 2c shows the top ten kinase-coding genes upregulated in the IPF samples. *DCLK1*, *PDK4*, *ERBB4*, *CDK1*, and *ribosomal protein S6 kinase A6* were upregulated by more than twofold (Fig. 2c). *STK33*, which is significantly upregulated in the integrated analysis, was also upregulated (log_2_ ratio 1.35), but with no statically significant difference. Furthermore, IHC revealed that DCLK1 and PDK4 proteins were mainly expressed in the epithelial layer and smooth muscle cells of fibrotic lesions in IPF lungs, whereas they were expressed in the airway epithelium of control lungs (Fig. 2d, e). Additionally, PDK4 expression was observed in the alveolar macrophages.

Of the 40 genes that had clinically available kinase inhibitors, only *ERBB4* was found to be significantly upregulated by more than twofold in IPF lung samples compared to the control lung samples (Fig. 2f). The ERBB4 protein had similar expression pattern to that of DCLK1 and PDK4 proteins, and it was mainly expressed in the epithelial layer and smooth muscle cells of fibrotic lesions in IPF lungs and in the airway epithelium of control lungs (Fig. 2g, Additional file 2: Fig. S2).

**Fig. 3:**
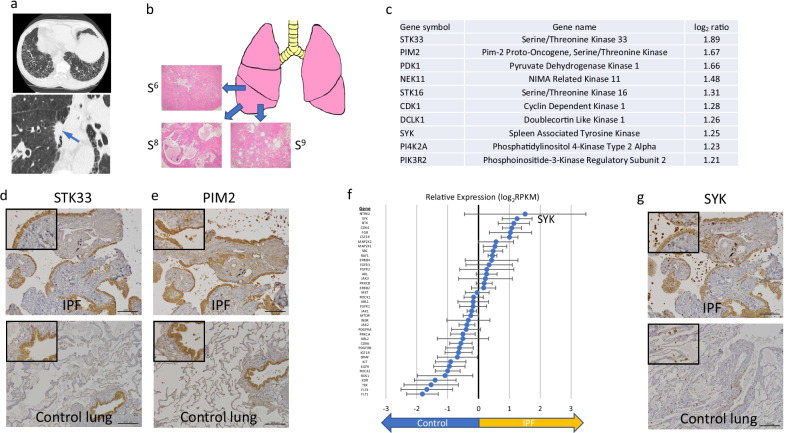
Clinical features and kinase expression profile of IPF lung tissues from case 2. **a** High-resolution computed tomography images of the lung. Blue arrow indicates lung cancer. **b** Sampling site and hematoxylin and eosin staining of each lung tissue. **c** Top ten upregulated kinase-coding genes in IPF lung samples compared to control lung samples. **d** Immunohistochemistry of STK33. Scale bar = 200 µm. **e** Immunohistochemistry of PIM2. Scale bar = 200 µm. **f** Expression profile of the 40 selected genes encoding kinases having clinically available kinase inhibitors. Error bars indicate standard error. **g** Immunohistochemistry of SYK. Scale bar = 200 µm

*Case 2:* A 67-year-old male with a smoking history (23.5 pack-years) who was diagnosed with lung squamous cell carcinoma (T2N0M0, stage IB) along with IPF. HRCT revealed lung cancer of the right lower lobe and bilateral honeycombing, indicating a UIP pattern (Fig. 3a). The patient underwent right lower lobectomy, and the pathological examination confirmed UIP in the lung tissue. Three samples were collected from the resected right lower lobe (Fig. 3b). Figure 3c shows the top ten genes upregulated by more than twofold in IPF lung tissues compared to control lung samples. *STK33* and *PIM2* were the top two genes based on fold change in IPF lung tissues. IHC indicated that STK33 protein was mainly expressed in the epithelial layer of fibrotic lesions in IPF, whereas it was observed in the airway epithelium of control lungs (Fig. 3d). Furthermore, PIM2 protein was mainly detected in the epithelial layer of fibrotic lesions, smooth muscle cells, and alveolar macrophages in IPF tissues, whereas it was expressed in the airway epithelium and alveolar macrophages in control lungs (Fig. 3e). Of the 40 genes having clinically available kinase inhibitors, *SYK*; *Bruton tyrosine kinase*; *cyclin-dependent kinase 4*; *FGR proto-oncogene*, *Src family tyrosine kinase*; and *colony-stimulating factor 1 receptor* were upregulated by more than twofold in IPF lung tissues (Fig. 3f). IHC indicated that SYK protein was mainly expressed in alveolar macrophages and epithelial layer of fibrotic lesions (Fig. 3g).

**Fig. 4:**
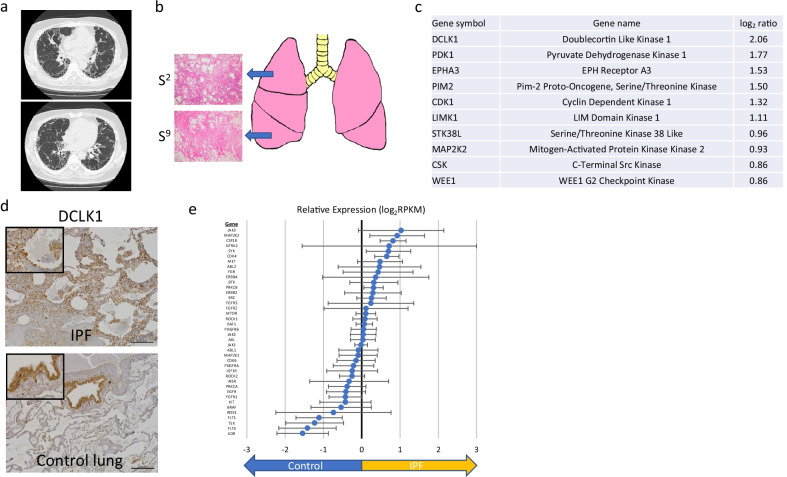
Clinical features and kinase expression profile of IPF lung tissues from case 3. **a** High-resolution computed tomography images of the lung. **b** Sampling site and hematoxylin and eosin staining of each lung tissue. **c** Top ten upregulated kinase-coding genes in IPF lung samples compared to control lung samples. **d** Immunohistochemistry of DCLK1. Scale bar = 200 µm. **e** Expression profile of the 40 selected genes encoding kinases having clinically available kinase inhibitors. Error bars indicate standard error

*Case 3:* A 61-year-old male with a smoking history (82.5 pack-years) who was diagnosed with IPF. HRCT revealed bilateral honeycombing, indicating a UIP pattern (Fig. 4a), and pathological examination confirmed UIP. The patient underwent a cadaveric transplant of the right lung. We obtained two lung samples from the right upper lobe and lower lobe (Fig. 4b). The top 10 upregulated kinase-coding genes are shown in Fig. 4c, with *DCLK1* being upregulated by more than twofold in IPF samples compared to the control lung samples. *STK33* was also upregulated (log_2_ ratio 1.30) with no statical significance. IHC indicated the expression of DCLK1 protein in the epithelial layer of fibrotic lesions and smooth muscle cells, similar to that observed in case 1 (Fig. 4d). Of the 40 genes having clinically available kinase inhibitors, *Janus kinase 3* was upregulated by more than twofold (Fig. 4e); however, the fold change was not statistically significant. In cases 4 and 5, no genes were upregulated by more than twofold in IPF lung samples compared to the control samples (Additional file 2: Fig. S3a, b).

## Discussion

The current study revealed a correlation between the gene expression signatures and degree of fibrosis, as assessed by Ashcroft score and indicated heterogeneity among IPF lung samples based on gene expression. In addition, we identified potentially targetable kinases, such as DCLK1, PDK4, ERBB4, STK33, PIM2, and SYK, which were overexpressed in IPF.

Our results demonstrated that *DCLK1* followed by *STK33* were the most upregulated genes in IPF lung tissues compared to control lung tissues. Consistent with our data, other studies have also reported the increased expression of these genes in IPF lungs [26–28]. Therefore, these genes may be universally upregulated in IPF lung tissues. DCLK1 regulates epithelial-mesenchymal transition (EMT) [29]. STK33 has been reported to be associated with cell proliferation as well as EMT in various cancer types [30]. In the current study, DCLK1 and STK33 proteins were expressed in the epithelial layer of fibrotic lesions in IPF lungs. Based on the evidence that epithelial cells differentiate into myofibroblasts through the EMT and that myofibroblasts promote lung fibrosis [31], DCLK1 and STK33 may serve as therapeutic candidates for IPF. In addition, recently, selective DCLK1 [32] and STK33 inhibitors [33] have been reported, which may provide alternative therapeutic strategies for IPF by suppressing the proliferation of aberrant epithelial cells and inhibiting EMT, thus hindering the progression of fibrosis.

Owing to the heterogeneous nature of IPF, its pathogenesis may vary in each patient. In some patients, IPF may be caused due to the dysfunction of AT2 cells, whereas in others it may be caused due to *MUC5B* gene aberration. Thus, the development of fibrosis and expression of genes may vary across individuals. In the present study, each patient showed a different gene expression pattern (e.g., case 1: DCLK1, PDK1, and ERBB4 expression; case 2: STK33, PIM2, and SYK expression). Except for *DCLK1* and *STK33*, these genes were neither identified by our integrated analysis nor found in the IPF Gene Explorer database, indicating that these genes are not universally expressed in IPF. However, these genes could serve as potential targets for personalized IPF therapy because they were uniquely upregulated in individual patients.

Among the selected genes having clinically available specific kinase inhibitors, ERBB4 was the most upregulated gene in IPF case 1. Dreymueller et al. reported that the release of inflammatory cytokines, such as CXCL8 and IL-6, from the smooth muscle cells was suppressed by inhibiting ERBB4 expression [34]. In addition, ERBB4 is reportedly associated with EMT in lung and gastric cancer cells [35, 36]. Therefore, ERBB4 could be a potential therapeutic target for IPF. Moreover, preclinical studies on SYK expression (upregulated in IPF case 2) have reported that its inhibition suppresses TGF-β1-induced myofibroblast activation and progression of fibrosis in the liver, kidney, skin, and lung [37–39]. Collectively, these results suggest that ERBB4 and SYK are attractive targets for IPF treatment; however, further preclinical studies are needed to confirm the suppression of lung fibrosis following the inhibition of the expression and activation of these kinases.

This study had several limitations. First is the small sample size, potentially leading to skewed results because of selection bias. Second, of the 21 samples analyzed in this study, 13 were obtained from residual specimens of lung cancer surgery, which included a relatively high proportion of patients with lung cancer. Although we microscopically confirmed that the samples for this analysis did not contain lung cancer cells, we cannot completely rule out the possibility that the lung tissue that develops cancer may bias our results. Further, IPF case 3 was treated with pirfenidone prior to tissue collection, which may have affected gene expression [40]. Third, we used bulk RNA sequencing analysis to explore the kinome expression profile in IPF lung tissues, as opposed to single-cell sequencing (scRNA-Seq). Unlike in scRNA-Seq [41, 42], the current study does not provide information on cell type-specific expression of the genes (e.g., fibroblasts and alveolar epithelium). Thus, our study results should be cautiously interpreted. However, the upregulation of the genes by RNA sequencing was confirmed at the protein level by IHC in the epithelial layer and smooth muscle cells of fibrotic lesions. We believe that future studies using scRNA-Seq will delineate the cell type-specific dynamic changes in the expression of genes during the process of fibrosis and identify better therapeutic targets. Fourth, to select a therapeutic target based on genetic profile, the part of the heterogeneous IPF lung that should be biopsied remains unclear. A large-scale integrated analysis with multiple patients in whom tissue sampling can be performed from each lobe of both lungs such as in case 1, may be able to provide clues regarding whether areas with strong fibrosis or weak fibrosis are more appropriate biopsy sites.

We performed a comprehensive kinase expression analysis using RNA sequencing to explore potential therapeutic targets for IPF and found that DCLK1 and STK33 may serve as potential candidates for molecular targeted therapy of IPF. In addition, PDK4, ERBB4, PIM2, and SYK might also be attractive targets in individual cases. Additional large-scale studies are warranted to develop personalized therapies for patients with IPF.

## Supplementary Information

**Additional file 1: Table S1.** List of 41 genes selected based on the signal-to-noise statistic. **Table S2.** Genes expressed differentially by more than twofold, in moderate and severe fibrotic samples compared to control lung samples. **Table S3.** List of 46 kinases having clinically available inhibitors.

**Additional file 2: Fig. S1.** a Clustering analysis of three samples from IPF case 2. b Clustering analysis of two samples from IPF case 3. c Signal-to-noise weighted-voting score based on 41 genes from IPF case 1. **Fig. S2.** Immunohistochemistry of ERBB4. Scale bar = 200 µm. **Fig. S3.** Expression of the 40 selected genes encoding kinases having clinically available kinase inhibitors in IPF cases 4 (a) and 5 (b).

## Data Availability

All data generated or analyzed during this study are included in this published article and its supplementary information files. The raw date of NGS datasets used and/or analyzed during the current study are available from the corresponding author on reasonable request.

## Abbreviations

IPF: Idiopathic pulmonary fibrosis
AT2: Alveolar type II
MUC5B: Mucin 5B
NSCLC: Non-small cell lung cancer
IHC: Immunohistochemistry
RPKM: Reads per kilobase per million mapped reads
DCLK1: Doublecortin-like kinase 1
STK33: Serine/threonine kinase 33
CDK1: Cyclin-dependent kinase 1
HRCT: High-resolution computed tomography
UIP: Usual interstitial pneumonia
PDK4: Pyruvate dehydrogenase kinase 4
ERBB4: Erb-B2 receptor tyrosine kinase 4
PIM2: Pim-2 proto oncogene, serine/threonine kinase
SYK: Spleen associated tyrosine kinase
EMT: Epithelial-mesenchymal transition
scRNA-Seq: Single-cell RNA sequencing

## References

[1] Nalysnyk L, Cid-Ruzafa J, Rotella P, Esser D (2012). Incidence and prevalence of idiopathic pulmonary fibrosis: review of the literature. Eur Respir Rev.

[2] Noble PW, Albera C, Bradford WZ, Costabel U, Glassberg MK, Kardatzke D, King TE, Lancaster L, Sahn SA, Szwarcberg J (2011). Pirfenidone in patients with idiopathic pulmonary fibrosis (CAPACITY): two randomised trials. Lancet.

[3] King TE, Bradford WZ, Castro-Bernardini S, Fagan EA, Glaspole I, Glassberg MK, Gorina E, Hopkins PM, Kardatzke D, Lancaster L (2014). A phase 3 trial of pirfenidone in patients with idiopathic pulmonary fibrosis. N Engl J Med.

[4] Richeldi L, Costabel U, Selman M, Kim DS, Hansell DM, Nicholson AG, Brown KK, Flaherty KR, Noble PW, Raghu G (2011). Efficacy of a tyrosine kinase inhibitor in idiopathic pulmonary fibrosis. N Engl J Med.

[5] Richeldi L, du Bois RM, Raghu G, Azuma A, Brown KK, Costabel U, Cottin V, Flaherty KR, Hansell DM, Inoue Y (2014). Efficacy and safety of nintedanib in idiopathic pulmonary fibrosis. N Engl J Med.

[6] Kim HJ, Perlman D, Tomic R (2015). Natural history of idiopathic pulmonary fibrosis. Respir Med.

[7] Ley B, Collard HR, King TE (2011). Clinical course and prediction of survival in idiopathic pulmonary fibrosis. Am J Respir Crit Care Med.

[8] Higo H, Kurosaki T, Ichihara E, Kubo T, Miyoshi K, Otani S, Sugimoto S, Yamane M, Miyahara N, Kiura K (2017). Clinical characteristics of Japanese candidates for lung transplant for interstitial lung disease and risk factors for early death while on the waiting list. Respir Investig.

[9] Tanaka S, Miyoshi K, Higo H, Kurosaki T, Otani S, Sugimoto S, Yamane M, Kiura K, Toyooka S, Oto T (2019). Lung transplant candidates with idiopathic pulmonary fibrosis and long-term pirfenidone therapy: treatment feasibility influences waitlist survival. Respir Investig.

[10] Sgalla G, Iovene B, Calvello M, Ori M, Varone F, Richeldi L (2018). Idiopathic pulmonary fibrosis: pathogenesis and management. Respir Res.

[11] Barkauskas CE, Cronce MJ, Rackley CR, Bowie EJ, Keene DR, Stripp BR, Randell SH, Noble PW, Hogan BL (2013). Type 2 alveolar cells are stem cells in adult lung. J Clin Invest.

[12] Zacharias WJ, Frank DB, Zepp JA, Morley MP, Alkhaleel FA, Kong J, Zhou S, Cantu E, Morrisey EE (2018). Regeneration of the lung alveolus by an evolutionarily conserved epithelial progenitor. Nature.

[13] Takezaki A, Tsukumo SI, Setoguchi Y, Ledford JG, Goto H, Hosomichi K, Uehara H, Nishioka Y, Yasutomo K (2019). A homozygous SFTPA1 mutation drives necroptosis of type II alveolar epithelial cells in patients with idiopathic pulmonary fibrosis. J Exp Med.

[14] Lawson WE, Grant SW, Ambrosini V, Womble KE, Dawson EP, Lane KB, Markin C, Renzoni E, Lympany P, Thomas AQ (2004). Genetic mutations in surfactant protein C are a rare cause of sporadic cases of IPF. Thorax.

[15] Seibold MA, Wise AL, Speer MC, Steele MP, Brown KK, Loyd JE, Fingerlin TE, Zhang W, Gudmundsson G, Groshong SD (2011). A common MUC5B promoter polymorphism and pulmonary fibrosis. N Engl J Med.

[16] Wollin L, Maillet I, Quesniaux V, Holweg A, Ryffel B (2014). Antifibrotic and anti-inflammatory activity of the tyrosine kinase inhibitor nintedanib in experimental models of lung fibrosis. J Pharmacol Exp Ther.

[17] Chaudhary NI, Roth GJ, Hilberg F, Muller-Quernheim J, Prasse A, Zissel G, Schnapp A, Park JE (2007). Inhibition of PDGF, VEGF and FGF signalling attenuates fibrosis. Eur Respir J.

[18] Du Z, Lovly CM (2018). Mechanisms of receptor tyrosine kinase activation in cancer. Mol Cancer.

[19] Hilberg F, Roth GJ, Krssak M, Kautschitsch S, Sommergruber W, Tontsch-Grunt U, Garin-Chesa P, Bader G, Zoephel A, Quant J (2008). BIBF 1120: triple angiokinase inhibitor with sustained receptor blockade and good antitumor efficacy. Cancer Res.

[20] Reck M, Kaiser R, Mellemgaard A, Douillard J-Y, Orlov S, Krzakowski M, von Pawel J, Gottfried M, Bondarenko I, Liao M (2014). Docetaxel plus nintedanib versus docetaxel plus placebo in patients with previously treated non-small-cell lung cancer (LUME-Lung 1): a phase 3, double-blind, randomised controlled trial. Lancet Oncol.

[21] Raghu G, Collard HR, Egan JJ, Martinez FJ, Behr J, Brown KK, Colby TV, Cordier JF, Flaherty KR, Lasky JA (2011). An official ATS/ERS/JRS/ALAT statement: idiopathic pulmonary fibrosis: evidence-based guidelines for diagnosis and management. Am J Respir Crit Care Med.

[22] Ashcroft T, Simpson JM, Timbrell V (1988). Simple method of estimating severity of pulmonary fibrosis on a numerical scale. J Clin Pathol.

[23] Golub TR, Slonim DK, Tamayo P, Huard C, Gaasenbeek M, Mesirov JP, Coller H, Loh ML, Downing JR, Caligiuri MA (1999). Molecular classification of cancer: class discovery and class prediction by gene expression monitoring. Science.

[24] Tomida S, Koshikawa K, Yatabe Y, Harano T, Ogura N, Mitsudomi T, Some M, Yanagisawa K, Takahashi T, Osada H, Takahashi T (2004). Gene expression-based, individualized outcome prediction for surgically treated lung cancer patients. Oncogene.

[25] Roskoski R (2020). Properties of FDA-approved small molecule protein kinase inhibitors: a 2020 update. Pharmacol Res.

[26] Nance T, Smith KS, Anaya V, Richardson R, Ho L, Pala M, Mostafavi S, Battle A, Feghali-Bostwick C, Rosen G, Montgomery SB (2014). Transcriptome analysis reveals differential splicing events in IPF lung tissue. PLoS ONE.

[27] IPF Gene Explorer. http://52.32.252.126:3838/geneExplorer/.

[28] Vukmirovic M, Herazo-Maya JD, Blackmon J, Skodric-Trifunovic V, Jovanovic D, Pavlovic S, Stojsic J, Zeljkovic V, Yan X, Homer R (2017). Identification and validation of differentially expressed transcripts by RNA-sequencing of formalin-fixed, paraffin-embedded (FFPE) lung tissue from patients with idiopathic pulmonary fibrosis. BMC Pulm Med.

[29] Sureban SM, May R, Lightfoot SA, Hoskins AB, Lerner M, Brackett DJ, Postier RG, Ramanujam R, Mohammed A, Rao CV (2011). DCAMKL-1 regulates epithelial-mesenchymal transition in human pancreatic cells through a miR-200a-dependent mechanism. Cancer Res.

[30] Kong F, Sun T, Kong X, Xie D, Li Z, Xie K (2018). Kruppel-like factor 4 suppresses serine/threonine kinase 33 activation and metastasis of gastric cancer through reversing epithelial-mesenchymal transition. Clin Cancer Res.

[31] Willis BC, duBois RM, Borok Z (2006). Epithelial origin of myofibroblasts during fibrosis in the lung. Proc Am Thorac Soc.

[32] Ferguson FM, Nabet B, Raghavan S, Liu Y, Leggett AL, Kuljanin M, Kalekar RL, Yang A, He S, Wang J (2020). Discovery of a selective inhibitor of doublecortin like kinase 1. Nat Chem Biol.

[33] Weiwer M, Spoonamore J, Wei J, Guichard B, Ross NT, Masson K, Silkworth W, Dandapani S, Palmer M, Scherer CA (2012). A potent and selective quinoxalinone-based STK33 inhibitor does not show synthetic lethality in KRAS-dependent cells. ACS Med Chem Lett.

[34] Dreymueller D, Martin C, Schumacher J, Groth E, Boehm JK, Reiss LK, Uhlig S, Ludwig A (2014). Smooth muscle cells relay acute pulmonary inflammation via distinct ADAM17/ErbB axes. J Immunol.

[35] Yu T, Li J, Yan M, Liu L, Lin H, Zhao F, Sun L, Zhang Y, Cui Y, Zhang F (2015). MicroRNA-193a-3p and -5p suppress the metastasis of human non-small-cell lung cancer by downregulating the ERBB4/PIK3R3/mTOR/S6K2 signaling pathway. Oncogene.

[36] Shi J, Li F, Yao X, Mou T, Xu Z, Han Z, Chen S, Li W, Yu J, Qi X (2018). The HER4-YAP1 axis promotes trastuzumab resistance in HER2-positive gastric cancer by inducing epithelial and mesenchymal transition. Oncogene.

[37] Qu C, Zheng D, Li S, Liu Y, Lidofsky A, Holmes JA, Chen J, He L, Wei L, Liao Y (2018). Tyrosine kinase SYK is a potential therapeutic target for liver fibrosis. Hepatology.

[38] Chen KH, Hsu HH, Yang HY, Tian YC, Ko YC, Yang CW, Hung CC (2016). Inhibition of spleen tyrosine kinase (syk) suppresses renal fibrosis through anti-inflammatory effects and down regulation of the MAPK-p38 pathway. Int J Biochem Cell Biol.

[39] Pamuk ON, Can G, Ayvaz S, Karaca T, Pamuk GE, Demirtas S, Tsokos GC (2015). Spleen tyrosine kinase (Syk) inhibitor fostamatinib limits tissue damage and fibrosis in a bleomycin-induced scleroderma mouse model. Clin Exp Rheumatol.

[40] Kwapiszewska G, Gungl A, Wilhelm J, Marsh LM, Thekkekara Puthenparampil H, Sinn K, Didiasova M, Klepetko W, Kosanovic D, Schermuly RT (2018). Transcriptome profiling reveals the complexity of pirfenidone effects in idiopathic pulmonary fibrosis. Eur Respir J.

[41] Xu Y, Mizuno T, Sridharan A, Du Y, Guo M, Tang J, Wikenheiser-Brokamp KA, Perl AT, Funari VA, Gokey JJ (2016). Single-cell RNA sequencing identifies diverse roles of epithelial cells in idiopathic pulmonary fibrosis. JCI Insight.

[42] Reyfman PA, Walter JM, Joshi N, Anekalla KR, McQuattie-Pimentel AC, Chiu S, Fernandez R, Akbarpour M, Chen CI, Ren Z (2019). Single-cell transcriptomic analysis of human lung provides insights into the pathobiology of pulmonary fibrosis. Am J Respir Crit Care Med.

