# Supplementary material for: Identification of targetable kinases in idiopathic pulmonary fibrosis
Source: Respir Res. 2022 Feb 7;23:20. doi: 10.1186/s12931-022-01940-y (PMC8822646; doi:10.1186/s12931-022-01940-y)
Supplement: Supplementary file 1 — Additional file 1: Table S1. List of 41 genes selected based on the signal-to-noise statistic. Table S2. Genes expressed differentially by more than twofold, in moderate and severe fibrotic samples compared to control lung samples. Table S3. List of 46 kinases having clinically available inhibitors. [file 12931_2022_1940_MOESM1_ESM.docx]

**Table S1** List of 41 genes selected based on the signal-to-noise statistic

**“induced” genes**

| No. | Gene | S |
| --- | --- | --- |
| 1 | ADCK4 | 1.31 |
| 2 | BMPR1B | 2.39 |
| 3 | CDK20 | 1.82 |
| 4 | DDR1 | 2.25 |
| 5 | DYRK1B | 1.26 |
| 6 | ERBB2 | 3.06 |
| 7 | ERBB3 | 1.39 |
| 8 | ERBB4 | 1.53 |
| 9 | FGFR2 | 1.89 |
| 10 | FGFR3 | 1.86 |
| 11 | HRAS | 1.35 |
| 12 | MAK | 1.89 |
| 13 | MAP3K19 | 1.81 |
| 14 | MAPK10 | 2.69 |
| 15 | MAPK15 | 1.96 |
| 16 | NEK1 | 2.67 |
| 17 | NEK10 | 1.94 |
| 18 | NEK11 | 3.08 |
| 19 | NEK5 | 1.37 |
| 20 | RIPK4 | 2.79 |
| 21 | RPS6KA6 | 1.77 |
| 22 | STK33 | 2.77 |
| 23 | STK36 | 1.79 |
| 24 | ULK4 | 2.90 |
| 25 | USP28 | 0.94 |
| 26 | WEE1 | 0.85 |

**“suppressed” genes**

| No. | Gene | S |
| --- | --- | --- |
| 1 | ACVRL1 | -1.28 |
| 2 | CDKL5 | -0.56 |
| 3 | CDKN2B | -1.06 |
| 4 | DDR2 | -0.48 |
| 5 | FLT1 | -0.98 |
| 6 | GATA3 | -1.92 |
| 7 | HSPB8 | -1.35 |
| 8 | ITK | -2.06 |
| 9 | LRRK2 | -4.16 |
| 10 | NPR1 | -1.72 |
| 11 | PRKCQ | -2.29 |
| 12 | ROR1 | -0.77 |
| 13 | ROS1 | -1.33 |
| 14 | STK17B | -2.16 |
| 15 | TEK | -1.46 |

**Table S2** Genes expressed differentially by more than 2-fold, in moderate and severe fibrotic samples compared to control lung samples

**Severe vs normal upregulated genes**

| Gene symbol | Gene name | Log_2_ ratio |
| --- | --- | --- |
| MAP3K19 | Mitogen-Activated Protein Kinase Kinase Kinase 19 | 4.41 |
| NEK10 | NIMA Related Kinase 10 | 3.65 |
| NEK5 | NIMA Related Kinase 5 | 2.94 |
| BMPR1B | Bone Morphogenetic Protein Receptor Type 1B | 2.59 |
| STK33 | Serine/Threonine Kinase 33 | 2.53 |
| MAK | Male Germ Cell Associated Kinase | 2.34 |
| MAPK15 | Mitogen-activated protein kinase 15 | 2.04 |
| NEK11 | NIMA Related Kinase 11 | 1.92 |
| DCLK1 | Doublecortin Like Kinase 1 | 1.87 |
| RIPK4 | Receptor-interacting serine/threonine-protein kinase 4 | 1.71 |
| ULK4 | Unc-51 Like Kinase 4 | 1.57 |
| COL1A1 | Collagen Type I Alpha 1 Chain | 1.55 |
| IDH1 | Isocitrate Dehydrogenase 1 | 1.54 |
| CDK1 | Cyclin-dependent kinase 1 | 1.49 |
| ERBB4 | Erb-B2 Receptor Tyrosine Kinase 4 | 1.41 |
| MAPK10 | Mitogen-activated protein kinase 10 | 1.33 |
| RPS6KA6 | Ribosomal Protein S6 Kinase A6 | 1.22 |
| CDK20 | Cyclin-dependent kinase 20 | 1.17 |
| STK16 | Serine/threonine-protein kinase 16 | 1.15 |
| PAK1 | P21 (RAC1) Activated Kinase 1 | 1.13 |
| MERTK | MER Proto-Oncogene, Tyrosine Kinase | 1.05 |
| DDR1 | Discoidin Domain Receptor Tyrosine Kinase 1 | 1.03 |
| IDH2 | Isocitrate Dehydrogenase 2 | 1,02 |

**Severe vs normal downregulated genes**

| Gene symbol | Gene name | Log2 ratio |
| --- | --- | --- |
| DAPK2 | Death Associated Protein Kinase 2 | -2.24 |
| FLT4 | Fms Related Receptor Tyrosine Kinase 4 | -2.10 |
| TEK | TEK Receptor Tyrosine Kinase | -2.08 |
| FLT1 | Fms Related Receptor Tyrosine Kinase 1 | -2.03 |
| CDKL5 | Cyclin Dependent Kinase Like 5 | -2.01 |
| CDKN2B | Cyclin Dependent Kinase Inhibitor 2B | -1.86 |
| NPR1 | Natriuretic Peptide Receptor 1 | -1.82 |
| FGFR4 | Fibroblast Growth Factor Receptor 4 | -1.75 |
| ROR1 | Receptor Tyrosine Kinase Like Orphan Receptor 1 | -1.56 |
| ACVRL1 | Activin A Receptor Like Type 1 | -1.51 |
| PKDCC | Protein Kinase Domain Containing, Cytoplasmic | -1.50 |
| KDR | Kinase Insert Domain Receptor | -1.44 |
| LMTK2 | Lemur Tyrosine Kinase 2 | -1.42 |
| MAP4K2 | Mitogen-Activated Protein Kinase Kinase Kinase Kinase 2 | -1.41 |
| PRKCQ | Protein Kinase C Theta | -1.34 |
| LRRK2 | Leucine Rich Repeat Kinase 2 | -1.30 |
| ROS1 | ROS Proto-Oncogene 1, Receptor Tyrosine Kinase | -1.28 |
| GATA3 | GATA Binding Protein 3 | -1.27 |
| GRK5 | G Protein-Coupled Receptor Kinase 5 | -1.24 |
| TIE1 | Tyrosine Kinase With Immunoglobulin Like And EGF Like Domains 1 | -1.22 |
| GAB1 | GRB2 Associated Binding Protein 1 | -1.17 |
| DDR2 | Discoidin Domain Receptor Tyrosine Kinase 2 | -1.13 |
| PRKCE | Protein Kinase C Epsilon | -1.11 |
| APC | APC Regulator Of WNT Signaling Pathway | -1.06 |
| FYN | FYN Proto-Oncogene, Src Family Tyrosine Kinase | -1.05 |
| PIK3C2B | Phosphatidylinositol-4-Phosphate 3-Kinase Catalytic Subunit Type 2 Beta | -1.05 |
| PEAK1 | Pseudopodium Enriched Atypical Kinase 1 | -1.04 |
| CDK11A | Cyclin Dependent Kinase 11A | -1.00 |

**Moderate vs normal upregulated genes**

| Gene symbol | Gene name | Log2 ratio |
| --- | --- | --- |
| DCLK1 | Doublecortin Like Kinase 1 | 1.67 |
| COL1A1 | Collagen Type I Alpha 1 Chain | 1.56 |

**Moderate vs normal downregulated genes**

| Gene symbol | Gene name | Log2 ratio |
| --- | --- | --- |
| DAPK2 | Death Associated Protein Kinase 2 | -1.41 |
| FLT4 | Fms Related Receptor Tyrosine Kinase 4 | -1.33 |
| FGFR4 | Fibroblast Growth Factor Receptor 4 | -1.25 |

**Table S3** List of 46 kinases having clinically available inhibitors

| gene symbol | gene name | drug |
| --- | --- | --- |
| CDK4 | Cyclin Dependent Kinase 4 | abemaciclib |
| CDK6 | Cyclin Dependent Kinase 6 | abemaciclib |
| BTK | Bruton Tyrosine Kinase | acalabrutinib |
| EGFR | Epidermal Growth Factor Receptor | afatinib |
| ERBB2 | Erb-B2 Receptor Tyrosine Kinase 2 | afatinib |
| ERBB4 | Erb-B2 Receptor Tyrosine Kinase 4 | afatinib |
| ALK | ALK Receptor Tyrosine Kinase | alectinib |
| MET | MET Proto-Oncogene, Receptor Tyrosine Kinase | cabozantinib |
| RET | Ret Proto-Oncogene | cabozantinib |
| TEK | TEK Receptor Tyrosine Kinase | cabozantinib |
| IGF1R | Insulin Like Growth Factor 1 Receptor | ceritinib |
| INSR | Insulin Receptor | ceritinib |
| ROS1 | ROS Proto-Oncogene 1, Receptor Tyrosine Kinase | crizotinib |
| BRAF | B-Raf Proto-Oncogene, Serine/Threonine Kinase | dabrafenib |
| NTRK1 | Neurotrophic Receptor Tyrosine Kinase 1 | entrectinib |
| NTRK2 | Neurotrophic Receptor Tyrosine Kinase 2 | entrectinib |
| NTRK3 | Neurotrophic Receptor Tyrosine Kinase 3 | entrectinib |
| MTOR | Mechanistic Target Of Rapamycin Kinase | everolimus |
| SYK | Spleen Associated Tyrosine Kinase | fostamatinib |
| FLT3 | Fms Related Tyrosine Kinase 3 | gilteritinib |
| AXL | AXL Receptor Tyrosine Kinase | gilteritinib |
| KIT | KIT Proto-Oncogene Receptor Tyrosine Kinase | imatinib |
| PRKCA | Protein Kinase C Alpha | midostaurin |
| PRKCB | Protein Kinase C Beta | midostaurin |
| PRKCG | Protein Kinase C Gamma | midostaurin |
| FGR | FGR Proto-Oncogene, Src Family Tyrosine Kinase | midostaurin |
| ROCK1 | Rho Associated Coiled-Coil Containing Protein Kinase 1 | netarsudil |
| ROCK2 | Rho Associated Coiled-Coil Containing Protein Kinase 2 | netarsudil |
| FLT1 | Fms Related Tyrosine Kinase 1 | nintedanib |
| KDR | Kinase Insert Domain Receptor | nintedanib |
| FLT4 | Fms Related Tyrosine Kinase 4 | nintedanib |
| FGFR1 | Fibroblast Growth Factor Receptor 1 | nintedanib |
| FGFR2 | Fibroblast Growth Factor Receptor 2 | nintedanib |
| FGFR3 | Fibroblast Growth Factor Receptor 3 | nintedanib |
| PDGFRA | Platelet Derived Growth Factor Receptor Alpha | nintedanib |
| PDGFRB | Platelet Derived Growth Factor Receptor Beta | nintedanib |
| CSF1R | Colony Stimulating Factor 1 Receptor | pexidartinib |
| ABL1 | ABL Proto-Oncogene 1, Non-Receptor Tyrosine Kinase | ponatinib |
| ABL2 | ABL Proto-Oncogene 2, Non-Receptor Tyrosine Kinase | ponatinib |
| SRC | SRC Proto-Oncogene, Non-Receptor Tyrosine Kinase | ponatinib |
| JAK1 | Janus Kinase 1 | ruxolitinib |
| JAK2 | Janus Kinase 2 | ruxolitinib |
| RAF1 | Raf-1 Proto-Oncogene, Serine/Threonine Kinase | sorafenib |
| JAK3 | Janus Kinase 3 | tofacitinib |
| MAP2K1 | Mitogen-Activated Protein Kinase Kinase 1 | trametinib |
| MAP2K2 | Mitogen-Activated Protein Kinase Kinase 2 | trametinib |
