# Supplementary figures and images for: Identification of targetable kinases in idiopathic pulmonary fibrosis
Source: Respir Res. 2022 Feb 7;23:20. doi: 10.1186/s12931-022-01940-y (PMC8822646; doi:10.1186/s12931-022-01940-y)

Supplementary figure 1

a

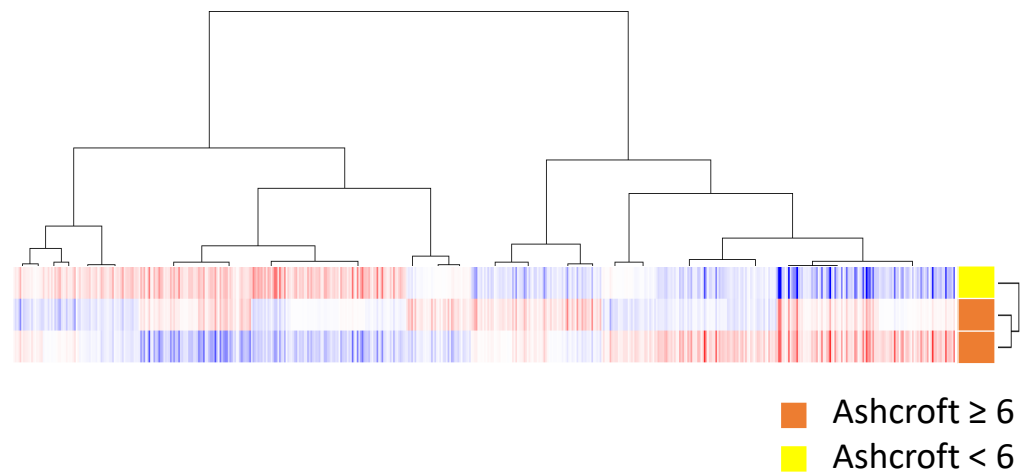

b

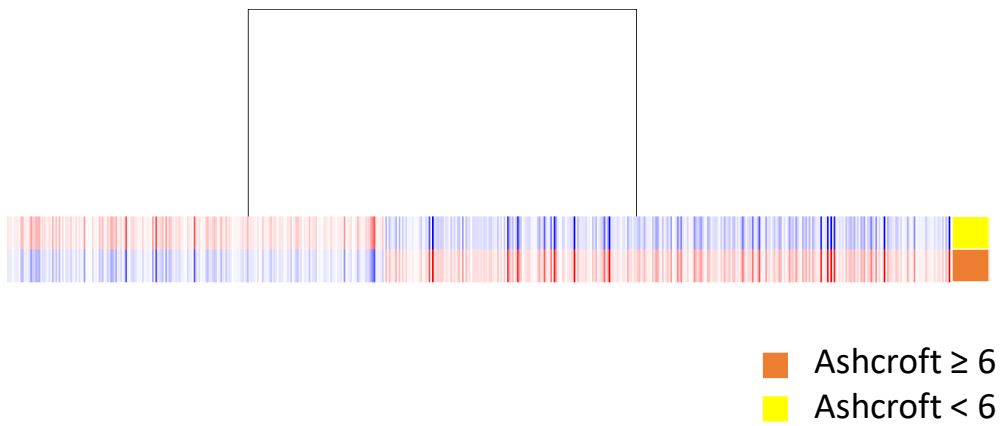

c

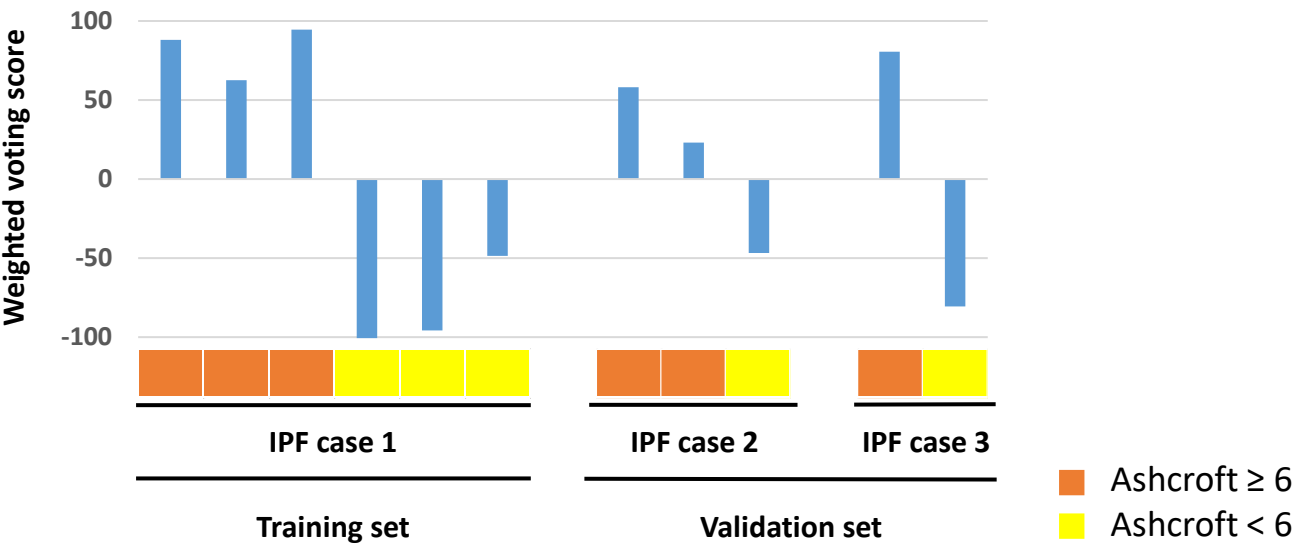

Supplementary figure 2

ERBB4

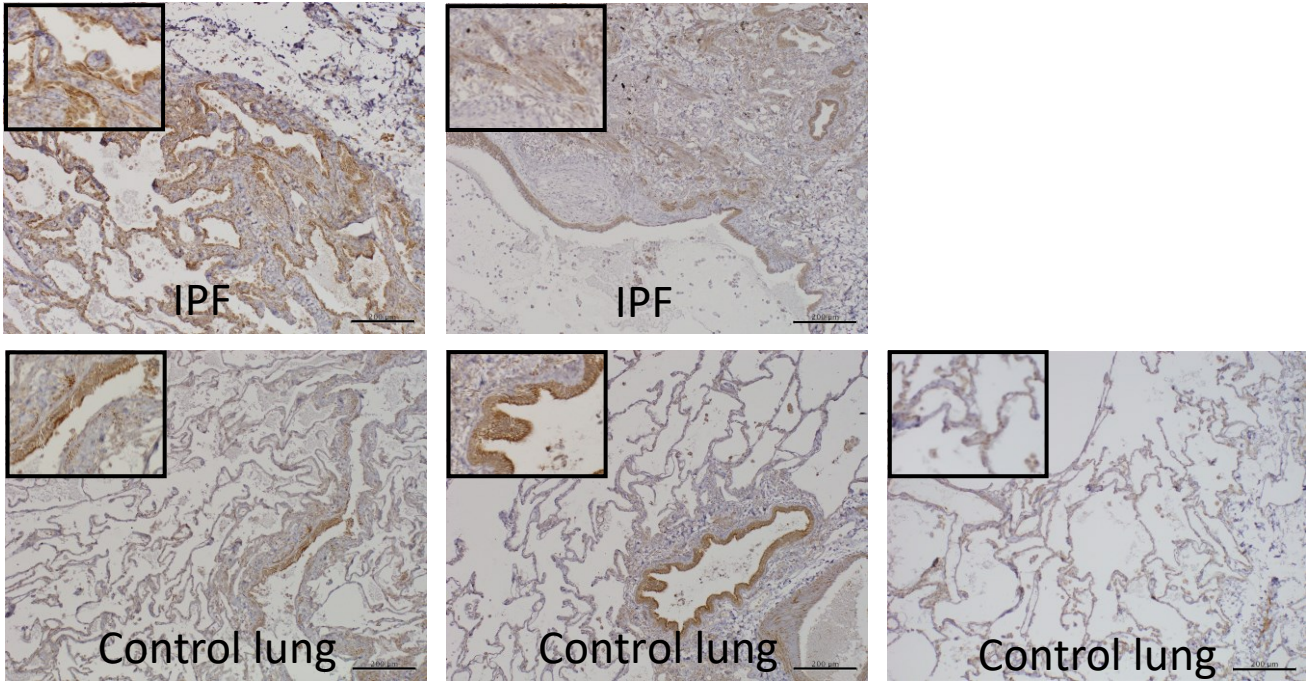

Supplementary figure 3

a

Case 4

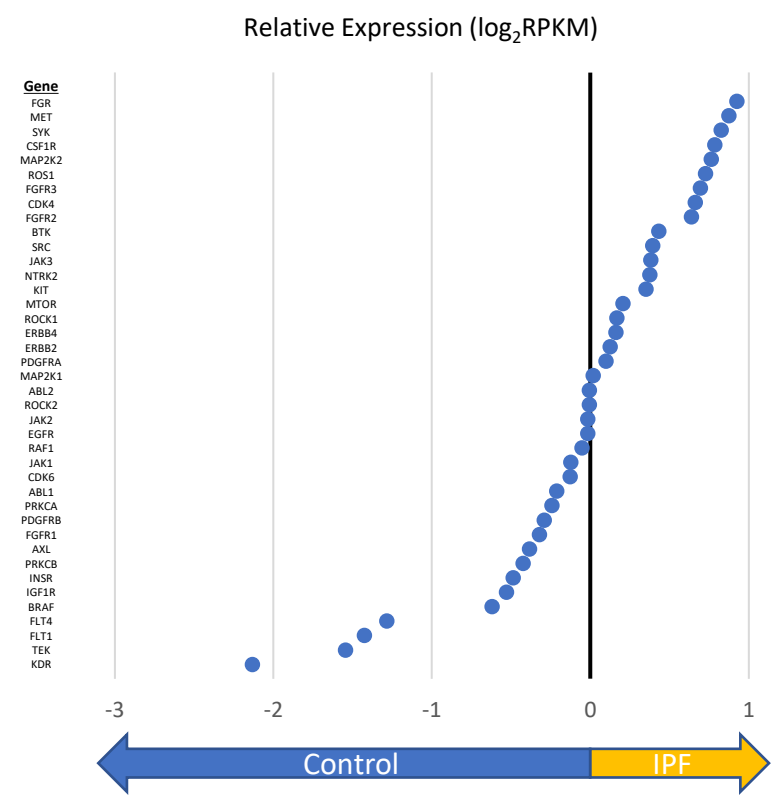

b

Case 5

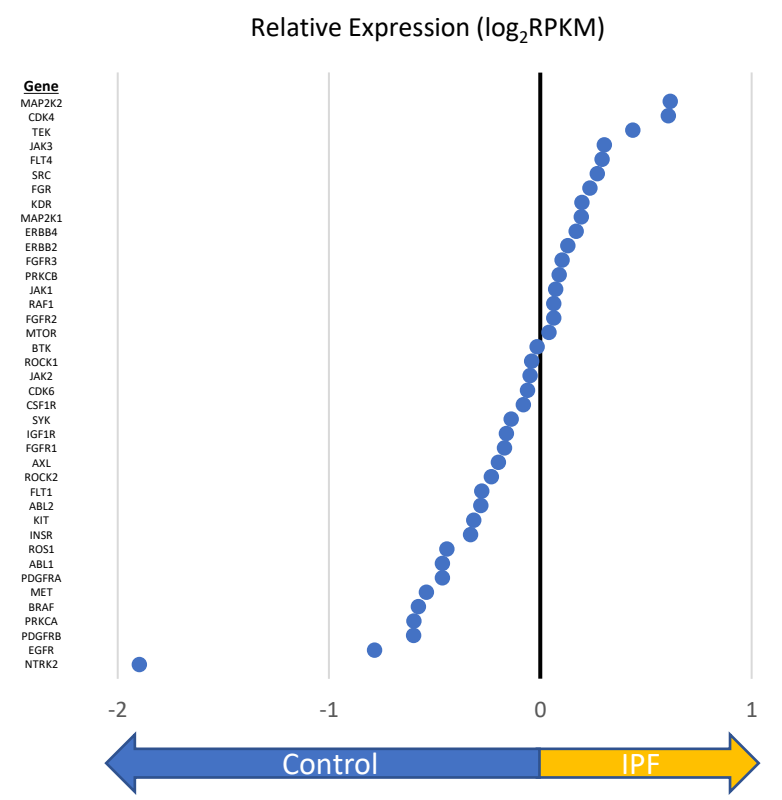

Supplement: Supplementary file 2 — Additional file 2: Fig. S1. a Clustering analysis of three samples from IPF case 2. b Clustering analysis of two samples from IPF case 3. c Signal-to-noise weighted-voting score based on 41 genes from IPF case 1. Fig. S2. Immunohistochemistry of ERBB4. Scale bar = 200 µm. Fig. S3. Expression of the 40 selected genes encoding kinases having clinically available kinase inhibitors in IPF cases 4 (a) and 5 (b). [file 12931_2022_1940_MOESM2_ESM.pdf]
